# Cleaner horizons: Exploring advanced technologies for pollution remediation

**DOI:** 10.1016/j.btre.2025.e00890

**Published:** 2025-03-28

**Authors:** Khadija Malik, Ashja Iftikhar, Quratulain Maqsood, Muhammad Rizwan Tariq, Shinawar Waseem Ali

**Affiliations:** Department of Food Sciences, Faculty of Agriculture Sciences, University of the Punjab, Lahore, Pakistan

**Keywords:** Pollution remediation, Advanced technologies, Environmental cleanup, Contaminant removal, Bioremediation

## Abstract

Soil pollution causes many harmful effects by its contaminants or pollutants, which are known as soil pollutants. They are causing serious problems in plants as well as in humans. By entering into plants, harmful chemicals become part of the food chain. When humans consume contaminated food, it has harmful effects on human health. Pollutants are making soil unfit for living. Many techniques are being used for the remediation of soil pollution. Some are traditional techniques; some are innovative and effective as emerging science and technology are going on. In this review, we have discussed some significant methods, their aspects, and how they are playing their role in the remediation. Biological methods such as living organisms, chemical, and genetic manipulation are modern techniques that are being used for soil pollution remediation. Genetic manipulations sometimes change the enzyme processes, which enhance the whole activity by changing some of the proteins of organisms related to enzymes. Pollution remediation can be done by the process of bio-augmentation, which uses different types of strains of microbes for treatment. As there is an increase in the formation of OH compounds, advanced oxidation technologies are being introduced to treat them. Trace metals and heavy metals are also a big problem for soil pollution, which can be treated by phytoremediation techniques that use many different strategies. Nanoparticles are also being used for the treatment of compounds like nitrates, manganese, arsenic, etc. This review will guide you through the different technologies for soil pollution remediation.

## Highlights

• Provides a comprehensive overview of modern techniques and technologies for soil pollution remediation.
• Emphasizes the role of emerging science and technology in shaping soil pollution remediation strategies.
• Discusses innovative remediation techniques, including genetic manipulation, bioaugmentation, advanced oxidation technologies, phytoremediation, and nanoparticle-based treatments.
• Offers practical guidance on the application of these technologies, including their effectiveness and potential challenges.

## 1 Introduction

Soil is a natural habitat of organisms, but the increasing industrialization, ¸urbanization, and exposure to chemicals are making this soil unfit for living, i.e. soil pollution. It poses a serious threat and is hazardous [1]. This is unavoidable; soil contaminants become part of the food chain when they enter the plants and then become part of food, ultimately affecting the human population [2]. Pesticides are generally applied to crops; only a few % of the pesticides are taken up by pests, and the rest becomes part of the soil, water, etc., and becomes part of the food and food chain [3]. Some metals can also cause serious effects when metals like arsenic (As), Chromium (Cr), and lead (Pb) stay in the sediments. From there, they become part of the food chain. They can also harm the fauna and flora present in the aquatic ecosystems [4]. The awareness about the hazardous effects of these chemicals on humans and the environment has increased, so it has been given attention internationally. The chemicals that are very critical and cause environmental pollution are the dumping of solid wastes, untreated industrial effluents, PCBs, persistent organic pollutants (POPs) like PAHs, and pesticides. These compounds cause environmental pollution by directly or indirectly releasing them into soil, water, or air. Soils contaminated with organic pollutants or inorganic like heavy metals are more dangerous as these become part of the food chain [5]. Contamination can harm humans directly, and the contaminants in marine life can affect humans indirectly by being part of the food chain [6]. Farmland and soil are polluted with heavy metals, and they form metalloids, which are non-degradable and are a threat to human health and food safety because they can only be transferred from one state to another. Metals are very tenacious in soil [7]. Volatilization can be caused by both organic and inorganic contaminants (Se, Ar, Hg) [8,9]. This review aims at identifying the sources, mechanisms and consequences of soil contamination risk with special reference to pesticides, heavy metals and POPs. It will attempt at pointing out how these pollutants find their way into the ground, become concentrated and into the food chain, posing enormous risks to human beings and other living creatures [10]. In addition, this review aims to discuss the contemporary approaches of soil remediation and to make an attempt to draw the attention to the necessity of coming up with eco-friendly prevention measures to prevent soil pollution and guarantee food safety.

### 1.1 Soil pollution: a growing threat

The resources present on the earth are overutilized by human-induced activities aggravated by environmental stress, which is the effect of changes in the climate, and they affect extreme weather events like heat waves, cold waves, heavy precipitation or storm events, such as tropical cyclones. Moreover, increased production, mobilization of trace elements, use and disposal of

**Fig. 1:**
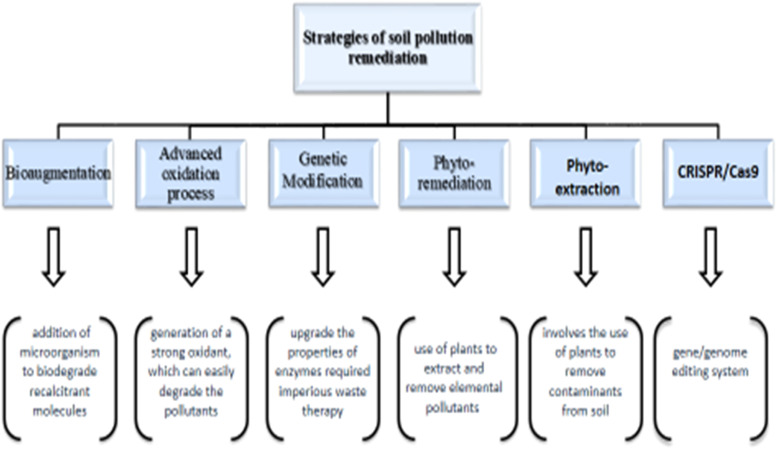
Strategies for soil pollution remediation.

Synthetic chemicals and other adverse human activities have contaminated soil; these are emerging as the biggest environmental reservoirs of pollution [11]. Soil pollution can be defined as the entry of foreign substances which can change the composition of soil chemically and physically. Sometimes, it occurs due to pesticides, fertilizers, and acid rain, which change the pH of the soil [12]. It is the demolition of the natural environment by the leaching of different chemicals or radioactive compounds, which increases the radioactive level of soil and delays analysis. The corruption of land, which destroys its chemical and biological states and affects it negatively directly or indirectly on humans, animals, or plants, is the pollution of agricultural land; they are caused by different agricultural pollutants like plant residues, weeds, burning of leaves, etc. [13]. Some techniques like phytoremediation, photooxidation, and phytovolatilization can be used to treat contaminants, as shown in Fig. 1. If they increase in concentration, they can cause toxicity in plants and can be dangerous to health [14].

### 1.2 Soil contaminants

The deposition of soil can be from geogenic and anthropogenic sources. Potentially toxic elements (PTEs) are caused by the weathering of parent materials [15]. Additionally, industrial contaminants are incorporated into agricultural practices themselves by fertilizers, pesticides, and sludge application. Soil works for the sink of pesticide contamination of groundwater [16]. Microplastics pose a threat to soil organisms if they change the composition of soil [17]. Heavy metals refer to some metalloids and metals having biological toxicity, i.e. cadmium, arsenic, lead, mercury, and chromium. They enter the soil agroecosystem through two types of natural processes derived from parent materials and through anthropogenic activities. Their pollution poses a significant threat to the health and well-being of organisms and human beings due to potential accumulation risk through the food chain. Heavy metals lessen the functional diversity of the soil microbial communities and disturb the soil's natural cycles [18]. Polycyclic aromatic hydrocarbons (PAHs) cause very high soil pollution, which is caused by incomplete combustion in heating plants and vehicles; industrial activities also cause it. Contamination can also be a result of agricultural activities like the use of pesticides. Chemical analyses are carried out to check the mutagenic and carcinogenic compounds formed by heavy metals, PAHs, and pesticides [19]. Polyaromatic hydrocarbons (PAHs) are a very large group of organic contaminants that are produced as a result of human-induced activities and natural activities. PAHs have carcinogenic, mutagenic, and teratogenic effects on human and animal health; consequently, PAHs have been accounted for as the most critical pollutants by the US EPA. The effects of PAHs on soil microbial activity and variation are mostly considered separately as single contaminants. At previously manufactured gas plants, PAHs frequently co-exist with other heavy metals [20].

## 2 Remediation technology

### 2.1 Conventional remediation techniques

#### 2.1.1 Physical remediation

It is believed that most physical remediation technologies have the advantages of simple equipment, easy operation, low cost and so on, and mainly include thermal desorption and soil replacement [21] but often generate large volumes of contaminated soil or waste materials that require proper disposal

#### 2.1.2 Chemical remediation

By injecting chemical remediation agents, the mobility, availability, and toxicity of heavy metals in soil are reduced through adsorption, precipitation, oxidation, reduction, polymerization, and complexation, which are known as chemical remediation [21]. The overall cost of treatment, including the cost of chemicals, equipment, and labour, can be significant

#### 2.1.3 Phytoremediation

Trace elements like Cu, Mn, and Fe are necessary for people, animals, plants, and microbes, as evidenced by the use of critical trace metals to increase agricultural yields [22]. Heavy metal (HM) pollution of soil has become a major environmental problem on a worldwide scale due to increased industrialization and agrarian practices [23]. Due to their affordability and viability, biotechnological treatments, such as phytoremediation, show growing potential for the removal and/or degradation of inorganic and organic pollutants from soils [24]. Using plants to absorb or break down pollutants in the soil and water and make them safe to use is known as phytoremediation [25]. The heavy metals that are already present in the soil are gathered and moved to the top sections of plants using this plant-based method [26].

##### 2.1.3.1 Techniques frequently employed in the phytoremediation of heavy metal-contaminated areas

In the phytoremediation of heavy metal-contaminated areas, the following technique or techniques are frequently employed:

• Phytoextraction
• Phytostabilization
• Phytovolatilization
• Hydraulic control or Rhizofiltration

##### 2.1.3.2 Phytoextraction

The process by which plant roots absorb and transport pollutants into shoots is known as phytoextraction. However, it is also sometimes referred to as phytoaccumulation, photoabsorption, or phytosequestration [27]. A plant with a high capacity for biomass production or pollutant accumulation should be utilized for phytoextraction [28]. With their extraordinary capacity to collect contaminants, hyperaccumulator species are the most suitable plants for phytoextraction. However, it is also possible to do phytoextraction with plant species that produce large biomass but have low accumulation rates [29]. Growing suitable plant species on site, gathering the metal-enriched biomass, and treating it to decrease its mass and size are the basic concepts of phytoextraction [30], as shown in Table 1.

**Table 1:** Associated pollutants/plant species, processes, and mechanisms of phytoremediation [6].

Phtotechnology	Phytoextraction	Phytostabilization	Phytovolatilization	Rhizofiltration
**Mechanism**	Hyperaccumulation in harvestable parts of plants	complexation, absorbtion and precipitation	volatilization by leaves through transpiration	Rhizosphere accumulation through sorption concentration and precipitation
**Plants**	*Brassica juncea, Thlaspi caerulescens, Helianthus annus*	*Brassica juncea*, Hybrid poplars, Grasses	*Arabidopsis thaliana, Brassica juncea*, Poplars, and Alfalfa	Corn, Rye, Spinach, Tobacco, *Helianthus annus*, and *Brassica juncea*
**Contaminants**	Co, Cr, Ni, Pb, Zn, Au, Mo, Hg, Ag, and Cd are inorganic. radionuclides: U, Sr, Cs, and Pb	Inorganic: Hs, Zn, Cd, As, Cu, and Cr	Organic/Inorganic: Chlorinated aqueous solutions, Inorganic: silver, mercury, as	Organic/Inorganic: Radionuclides such as Cd, Cu, Ni, Zn, and Cr metals

##### 2.1.3.3 Phytostabilization

Phytostabilization lowers the bioavailability of heavy metals (HM) in contaminated soils by using plants that are tolerant of metals [50]. The viability of phytostabilizing zinc-smelting slag by means of four woody plants combined with organic supplements. Research has demonstrated that cultivating plants directly in zinc smelting waste slag increased nutrient accumulation and decreased the bioavailability of heavy metals (Cu, Zn, and Cd) [31].

Phytostabilization is an *in situ* technique of remediating contaminated soils and is considered to be the most effective for the immobilization of substantially toxic heavy metals through rendering them immobile and no longer available for leaching into the water table of ground water or into the food chain [32]. Compared to other phytoremediation techniques that comprise phytoextraction or phytovolatilization, phytostabilization aims at immobilizing the contaminants. The following mechanisms are involved: root adsorption, which is a physical process through which contaminants stick on the root surface or are adsorbed by the cell wall; precipitation in which root exudates change the pH or redox status of the contaminants and induces their precipitation in effectively immobile forms [33]. In addition, plants play their part in the stabilization process by releasing extra organics through what is referred to as root exudate, which also immobilized the metals by forming complexes with it. Furthermore, vegetation results in the reduction of erosion and minimizes the spread of pollutants through wind or water borne system and, therefore, is considered a cheap ecological method of handling contaminated soil. Several investigations have proved that phytostabilization can be applied successfully to control lead (Pb), arsenic (As) and cadmium (Cd) contaminated soils by using vetiver grass (Vetiveria zizanioides) and poplars (Populus spp.) [34]. It has received much attention as a cost effective and efficient method of land reclamation and remediation of mining and industrial effected sites.

##### 2.1.3.4 Phytovolatilization

Phytovolatilization is a phytoremediation procedure that uses plants to absorb harmful elements from the soil and change them into less dangerous volatile forms. The changed forms are then released into the atmosphere by transpiration through the leaves or foliage system of the plant. This method may be used to detoxify some heavy metals, including selenium, mercury, and arsenic, as well as other organic contaminants [35].

With reference to heavy metals, plants absorb metal ions, metabolize them into less toxic forms of volatile elements and discharge them into the atmosphere. For example, Hg may be adsorbed in the forms of Hg(II) and then transported to the root cell where it is then reduced to elemental Hg [Hg(0)] then evaporated [36]. This process remove mercury or decrease its concentration in the soil and help to avoid possible negative impact for the terrestrial and aquatic environment. For instance, selenium (Se) can more or less be taken up in forms of selenate or selenite, converted into compound called dimethylselenide, which is a volatile selenium and then expelled into the air. It has some advantages such as reduction in the problem of removal of contaminated plant biomass which is present in other phytoremediation forms such as phytoextraction [37]. Furthermore, the technique is non-destructive and cheaper when compared to others applicable for large scale use across areas with pollutants. Nevertheless, a major limitation is the possibility of reinjecting contaminants through air into the atmosphere this may call for precautions to minimise other adverse impacts on the environment.

For instance, numerous studies have been carried out on generic ability of different plant species as concerns phytovolatilization.The bioaccumulation of Hg(II) and its reduction to Hg(0) when plants engineered with the bacterium merA gene were used to transmute this compound through phytovolatilization [38]. Terry and Zayed [39] studied the effects of selenium phytovolatilization with plants such as Brassica juncea, and it was shown that the release of volatile selenium compounds resulted in a decrease of selenium content in the soil. However, when applied, phytovolatilization should take into count the conditions at the site and the properties of pollutants in question. For example, it is most useful in those cases where there is a bioavailability of contaminants in the soil and where plants can metabolise these contaminants into volatile substances [40]. Moreover, more studies are required to identify how the atmospheric emissions of pollutants can be controlled so that this technology meets the objectives of the study and is safe for the environment.

##### 2.1.3.5 Rhizofiltration

Rhizofiltration uses plant roots to adsorb contaminants in the surrounding root zone (rhizosphere), concentrating and precipitating them on or within the root [41]. Root exudates (secondary metabolites) released by plants' roots will undergo biogeochemical processes that precipitate contaminants on the roots or into the water body. Then, adsorption of contaminants on the roots, within the roots, or translocation to the phyllosphere (plant organs above-ground) will occur according to the type of plant, contaminant species, and concentration [42]. Plants with fibrous roots could have greater efficiency in hemofiltration as fibrous roots have a larger surface area exposed to the contaminants, which will increase the rate of accumulation [43]. In this phytoremediation method, radionuclides (U, Cs, Sr) and metals (Cd, Cr, Cu, Ni, Pb, and V) are removed [44].

### 2.2 Advanced remediation approaches

#### 2.2.1 Advanced oxidation process

**Fig. 2:**
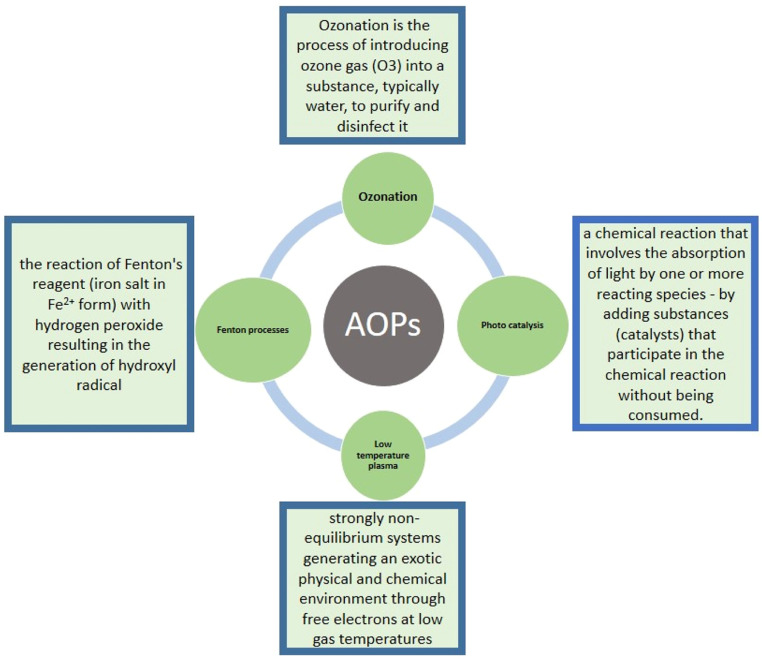
Processes of advanced oxidation.

The versatility of AOPs is also raised because they offer different processes for OH generation, allowing a finer difference in the specific treatment requirements. The techniques that are most often used in AOPs are (i) Fenton oxidations, (ii) plasma oxidation, (iii)) photo-catalysis, and (iv) ozonation [45]. A lot of work has been published on the remediation of soil pollution. Many approaches have been devised to remove contaminants and take them up to an acceptable limit [46]. One of the ways is chemical oxidation, which has the possibility of immediately treating soils that are polluted with toxic and bio-refractory organic compounds [47]. After washing, the wastewater has a more excellent content of organic contaminants and metals, which needs further treatment before discharge. Generally, metals are easy to grasp by alkali precipitation in wastewater (such as lime); moreover, it is a crucial step in which organic contaminants need biological treatment or chemical oxidation for the application of a chemical-enhanced washing process (Fig. 2).

Advanced oxidation based on sulfate radicals (SO_4_^−^, E^0^ = 2.6 V) has many advantages like stability, high efficiency, simple operation, and no secondary pollution. It induced substantial interest on the grounds of the decomposition of hazardous compounds in the aqueous phase or soil [48]. Chemical oxidation is used to mineralize pollutants such as carbon dioxide (CO_2_), water (H_2_O), and inorganics or to transform them into harmless or biodegradable products [49]. AOPs gained much attention, and they are taken into account because they are environment friendly and for organic contaminated soil pollution [48]. The following oxidants are used: permanganate (MnO_4_^−^), hydrogen peroxide (H_2_O_2_), ozone (O_3_), persulfate (S_2_O_8_^2−^), and Fenton process (OH). For the formation of hydroxyl radicals (OH, E^0^ = 2.8 V) hydrogen peroxide (E^0^ = 1.8 V) is generally applied with iron species (Fe^2+^, Fe^3+^, Fe_2_O_3_, Fe_3_O_4_ and Fe^0^) [50,51].

Studies have shown when the ratio of C/N/P is regulated to 100/5/1, 100/10/1, and 100/15/1, the activity of bacteria for bio-stimulation practice is bearable to many hydrocarbons and can be used as a source of carbon for their growth [52]. The optimum C/N/P ratio is very contrasting for remediation as different characteristics of polluted soil have various microbial communities. The standard formula for bio-stimulation strategy, which is commonly accepted is normally a ratio of C/N/P of 100/10/1 [53]. So, Bioaugmentation is the best option for soil that is contaminated with low Indigenous populations of hydrocarbon degraders [54]. Moreover, bio-augmentation provides a suitable out-turn [55]. It has the benefit that when particular microbial degraders are inaugurated, the degradation procedure can be initiated abruptly.

The effects of bioaugmentation are case-specific and controversial, as bioaugmentation technology has been dictated to be an adequate method for improving the degradation of petroleum in soil. Some studies designate that bioaugmentation is a sole degradation efficiency temporarily, and to get effective petroleum decontamination, biostimulation reappears as an ideal remediation strategy [55,56].

#### 2.2.2 Electrokinetics remediation

The electrokinetic remediation technique may be used to remove the contaminated material from the soil by employing electromigration, electropercolation, and electrophoresis in a low-density DC electric field. Electrokinetic remediation (EKR) shows significant potential for the removal of heavy metals from low-permeability soil due to its cost-effectiveness, efficiency, and ecologically benign nature [57]. Pollutants in the electrode zone become more abundant when charged ions or soil particles move in a targeted manner under the influence of an electric field [58].

An investigation of the efficacy of electrokinetic remediation (EK) in carbonaceous soils was conducted in 2017. The study showed that petroleum pollutants may be successfully removed from carbonaceous soil by electrokinetic remediation employing graphite electrodes and Na2SO4 as electrolytes. The study's findings offer important new information on the ideal operating parameters for successfully using the EK approach for soil remediation [59]. Furthermore, the work by Parameswarappa et al. showed that hydrocarbon pollutants may be successfully removed from fine-grained soils with a high clay concentration using electrokinetics (EK) in conjunction with a nonionic surfactant. EK obtained an outstanding clearance rate of 80 %, which was higher than hydraulic flushing's effectiveness of 52 % when the surfactant content was the same. This study emphasizes the potential of EK as a useful remediation method for dealing with hydrocarbon contamination in difficult-to-treat soil types [60].

The EK technique is widely applied in organochlorine cleanup. Suanon removed less than 10 % of the OCs from the polluted soil using a single-EK remediation procedure at a voltage of 2 V cm after 7 days of remediation [61].

To clean up soil polluted with chromium, EKR is used in conjunction with exchange resin adsorption. Exchange resin extracted chromium from polluted soil more effectively than standard EKR and required no additional electricity [62]. Numerous investigations verified that when the applied voltage gradient increases during an electrochemical reaction (EKR), so does the rate of Cd elimination. Additionally, the elevated pH causes Cd to concentrate in the area of the cathode. Citric acid can improve the efficiency of metal extraction, including the extraction of Cd, Cr, and other elements, as several studies have shown.

By using traditional electrokinetic remediation, 57 % of the original Cr and 49 % of the initial Cd were removed. With a 96 h polarity exchange interval, the new polarity exchange technique eliminated 82 % of Cd and 70 % of Cr, increasing the removal of heavy metals and bringing the pH of the soil down to 5–7. By using the polarity exchange technique with a 48 h polarity exchange interval, 88 % of total Cr and 94 % of total Cd were eliminated [63].

#### 2.2.3 Bioremediation and genetic modification in pollutant degradation

bioremediation is the use of living organisms, primarily microorganisms, to degrade the environmental contaminants into less toxic forms.The majority of pollutants that are resistant to typical native bacteria can be effectively removed by the modified microorganisms [64]. The researchers have proposed several strategies, including modifying the specificity and affinity of enzymes and introducing modifications to genes and regulatory pathways [65]. Microbial communities can usually evolve certain specific function genes under the stress of heavy metals (HMs). Examples of these genes are the czc gene cluster (Cd), cop gene cluster (Cu), aox gene cluster (As), and mer gene cluster (Hg), which can support microbial survival in some extremely harsh environmental situations. Genes required for pollutant degradation can aid in the accumulation, transformation, and detection of HMs [65]. Several essential enzymes, including monooxygenase, dioxygenase, dehydrogenase, and hydro-aldolase, are required for the degradation of PAHs by GEMs. Several different enzymes must interact for PAHs to be broken down into simpler substances, and the interactions between these enzymes are crucial to the process [66]. For instance, the enzymes required for the conversion of naphthalene to salicylate in the upper pathway from pseudomonas stutzeri AN10 are encoded by the nahAaAbAcAdBFCED gene. In contrast, the enzymes needed for the conversion of salicylate to pyruvate and acetyl-CoA in the lower pathway of naphthalene degradation are encoded by the nahGTHINLOMKJ gene. As a result, these functional genes are frequently utilized to create GEMs that have PAH-degrading capabilities [67].

Recent studies have revealed that the metabolism of benzo alpha pyrene, the primary component of polycyclic aromatic hydrocarbons (PAHs), requires the aspergillus bapA gene, which produces P450 monooxygenase [67]. Six fungal and seven bacterial native strains were assembled to form a microbial consortium to clean up PAHs. This consortium showed a high level of tolerance to phenanthrene, pyrene, and benzo(a)pyrene, and it was able to use PAHs as the only source of carbon.10 % more PAHs were degraded when two genetically modified fungal strains were added to the consortium. These strains produced ligninolytic enzymes that were very effective for the first oxidation of PAHs [67]. Fenamiphos Hydrolyzing Enzyme (FHE), a phosphotriesterase type enzyme from Microbacterium esteraromaticum, has been used to break down the neurotoxic pesticide Fenamiphos. P-O-C bonds in phenamiphos are specifically broken by the FHE enzyme, creating a variety of intermediate molecules. Methyltransferases then convert them into amino acids [67] (Table 2).

**Table 2:** Genetically engineered bacteria for remediation of heavy metal [68].

Heavy Metal	As	Cd²+	Cr⁶+	Cr	Hg	Hg	Ni	Hg
**Initial Concentration (ppm)**	0.05	-	1.4–1000	-	-	7.4	145	-
**Removal Efficiency (%)**	100	–	100	-	-	96	80	-
**Genetically Engineered Bacteria**	*E. coli* strain	*E. coli* strain	Methylococus capsules	P.putida strain	*E. coli* strain	*E. coli* JM109	P.fluorescens 4F39	Achromobacter sp AO22
**Expressed Gene**	Metallaoregulatory protein ArsP	SpPCS	CrR	Chromate reductase (ChrR)	Organomurcurial lyase	Hg²+ transporter	Phtochelatin synthase (PCS)	mer

#### 2.2.4 Challenges in implementing GMOs for soil remediation

To determine how a microbial consortium or the use of genetically engineered microbes in the field would affect the native soil microbiome, ecological risk assessment is an essential procedure [69]. Although there are many harmful pollutants, only a few of them can be broken down by living things since we don't know enough about the right enzymes and catabolic pathways. Finding new bacterial species may open the door to the development of new enzymes and metabolic pathways, but it's still challenging to isolate and grow natural bacteria [70]. Horizontal gene transfer risk is the main danger associated with GMOs. It is the uptake of genes by microbes under any ecological environment by conjugation, transduction, or transformation. This happens during ecological shifts and facilitates microorganisms' uptake of inherited genes. In living things, genes derived from genetically modified organisms (GMOs) may create health problems. In the absence of appropriate measures, the genes would propagate to native strains [71]. Numerous microbial organisms used in bioremediation may be detrimental. *Burkholderia cepacia*, for example, can bioremediate harmful nitro-compounds; however, it has also been linked to multiple antibiotic resistances and is the causal agent of human cystic fibrosis. The U.S. EPA expressed reservations about its usage as an environmental protection agent because of this [72]. One issue that concerns both the public and scientific groups is the containment of recombinant microorganisms after the bioremediation process is completed to prevent unwanted environmental effects [73].

#### 2.2.5 Phycoremediation

The technique known as "phycoremediation" uses macro- or microalgae to extract both harmful and non-toxic substances from solid, liquid, or gaseous wastes or to sequester carbon dioxide (CO2). The organisms do this by absorbing, accumulating, or transforming substances [74]. The ability to grow both heterotrophically and autotrophically, high surface area/volume ratios, tolerance to HMs, phototaxy, the capacity for genetic manipulation, and the expression of phytochelatins are some of the traits that make algae ideal candidates for the selective removal of HMs.The phycoremediation process, which lowers nutrients and heavy metals to the lowest possible levels, typically lasts three to twenty-six days. *Chlorella vulgaris, Chlorella variabilis, Scenedesmus obliquus* and *Scenedesmus abundans* as well as *Chlamydomonas reinhardti* have proved to be effective in HM remediation [75].

#### 2.2.6 Bioaugmentation: a promising approach for pesticide degradation

The addition of competent strains or association of microorganisms from the contaminated matrix to speed up the degradation rate of the soil is known as bioaugmentation [76,77]. The main focus is that the exogenously enhanced genetic variety will raise the metabolic proportion of the microbial community, which is present previously in biotopes that were scheduled for cleanup, which, as a result, leads to a broader stock of productive biodegradation processes [78].

**Fig. 3:**
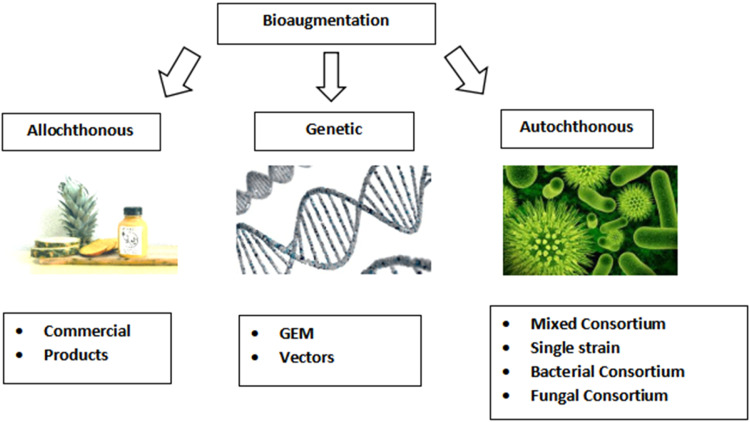
Types of bioaugmentation as one of the engineered bioremediation strategies [79].

The increased use of pesticides and the awareness about their harms make it possible to limit their use or to introduce less harmful pesticides. Two techniques can serve this: biostimulation and bioaugmentation. The last one is more reliable for the removal of pesticides and their residues (Fig. 3).

#### 2.2.7 CRISPR/Cas9 technology and waste management

Phytoremediators, or plants that do phytoremediation are subject to a number of limitations, such as poor biomass output and a sluggish growth rate, metal selectivity, and varying environmental growth circumstances [80]. Genome editing is a viable technique for changing our plant's expression of a desired gene [81]. The CRISPR technique was initially applied in 2012, and in 2013 it was employed in plants [82]. It has become increasingly sophisticated in the field of plant sciences [57], surpassing previous methods like precision editing by HDR [59] and targeted mutagenesis [58]. The viability of using CRISPR-assisted genome editing technology for the phytoremediation of heavy metals and metalloids has been tested. Plant changes made possible by CRISPR aid in withstanding, immobilizing, and stabilizing a variety of contaminants. Numerous phytotechnologies, including phytoremediation, have intriguing prospects due to the accuracy, economy, and potential of CRISPR-mediated gene editing [83]. In addition to making pollutants more amenable to phytoremediation, CRISPR techniques can be utilized to understand better the mechanisms underpinning pollution accumulation, evaporation, and destruction [84].

Its two components are a guide RNA (gRNA), which guides the Cas9 nuclease to a particular area of the genome, and a DNA double-strand break (DSB) that is produced. Gene deletion may result from a cell's endogenous repair mechanism [85]. CRISPRa (activation) is a method that has been effectively applied to plants and can change the expression of a gene up to 1000 times [86]. By attaching a transcriptional activator peptide to a catalytically dead dCas9, the CRISPRa method can increase the transcription of a certain gene by directing the dCas9-activator to the target gene's promoter or regulatory regions using a specifically created gRNA sequence [87].

Using a rice gene called OsMyb4, which encodes the Arabidopsis thaliana COR15a pressure regulator, transgenic spring canola (Brassica napus L.) was produced from wild-type canola (Brassica napus L.) [65]. The ability of tobacco and Arabidopsis plants to tolerate and accumulate metals such as Cd, Cu, and Zn was observed to be enhanced when the metallothionein-encoding genes were expressed [66]. Through CRISPR-mediated control of the metal transporter gene OsNRAMP5, Cd buildup in indica rice was reduced without compromising yield [88]. Using the CRISPR/Cas system to handle heavy metal stress in plant species has the potential to enhance plants' capacity for phytoremediation [89]. Plant genomes that are suited for phytoremediation can be altered by CRISPR/Cas9, as demonstrated by the ability to modify the genomes of poplar and maize, for example [90]. The complex genome of maize can be altered using the CRISPR/Cas9 technique to absorb more significant metal concentrations despite its high ploidy. This is advantageous because maize can produce a sizable amount of biomass [91].

Plant vacuoles store HMs and preserve the homeostasis of HMs. Therefore, modifying the vacuolar transporter could help increase the essential HMs and decrease the non-essential HM in the crop's edible section. It may also encourage the build-up of non-essential heavy metals in non-crop plants. For example, NtNRAMP3, a vacuolar localized membrane transporter, regulates the subcellular distribution of Cd in tobacco plants (vacuoles to the cytoplasm). NtNRAMP3 gene deletion by CRISPR/Cas9 was observed to hinder Cd translation from the vacuole to the cytosol in tobacco leaves as compared to the wild type. According to this finding, the vacuolar Cd concentration was much higher than that of the tobacco plants grown in the wild. Consequently, because of the functional loss, vacuolar storage of Cd increases the effectiveness of phytoremediation [92].

#### 2.2.8 CRISPR-mediated phytoremediation strategies

Many biotechnological techniques and strategies are used for the phytoextraction or phytoremediation of toxics especially the heavy metals. This dialogue includes synthetic biology approaches for increasing the stress tolerance of legumes and additional useful crops to heavy metals and demonstrating several cutting edge instances [88]. All such methods employ synthetic biology to alter the characteristics of plants in order to enable them to better withstand contaminants. Among these, one can distinguish the CRISPR technique that implies multiple gene or transcription factories regulation by gene knockout or knockdown techniques. Nutrient adequacy and the maintenance of balanced cellular ions and osmotic potential stands out as one of the major functional correlates of plant longevity. Tolerance to heavy metal stress becomes a paramount consideration in view of the fact that plants are subjected to metal toxicity, and therefore, there is a need for alteration of increased levels of variety that embrace physiological, biochemical and molecular changes [93]. Partial resistance against these conditions is a factor influenced by genotype as well as the environment where the plants are grown. In recent years, detailed advancements have been achieved in the study of several regulatory processes that allow the plant to withstand or tolerate the heavy metal stress and at the same time, exhibit non-toxic symptoms of growth.

This section discusses some of the recent developments in the biotechnological tools such as the functions of microRNAs (miRNAs), plant growth-promoting rhizobacteria (PGPR), and the new information on the part played by salicylic acid (SA) and nitric oxide in the plant ability to counter the effects of heavy metals. Contaminated soil and water are a great threat to the environment and human health. But currently, there are new techniques that stand a better chance to manage these risks in a safer way that is known as phytoremediation [94]. They include the uptake, translocation and bioaccumulation/toxicity of the toxic metals in plants. This technology is cheaper than most other remedial measures because plants take up heavy metals from the environment and incorporate them into their structures. To enhance this process, coping techniques have been established which encompasses both the phsysiological and molecular mechanisms of the plants [95]. Further, a few field treatments have also been carried out independently to investigate the efficacy of some plant species for phytoremedial applications.

#### 2.2.9 CRISPR/Cas9 technology for the phytoremediation of heavy metals

Mineral nutrition of plants depends both on genetical predisposition and on the characteristics of the soil and its nutritive properties. Due to their feeding strategy, plants must be more specific in their feeding habits in that they must be able to feed optimally while at the same time not allowing toxic levels of heavy metals to interfere with their metabolism [84]. Public awareness of plants’ application in the purification of soils affected by pollution has been well adopted. Plant and their symbiotic bio partners may have a central role to ensure the bioremediation of the contaminated soils. Phytoremediation measures may include the wild plant-microbe associations or specific and special methods of planting and growing plants. The researchers are struggling to develop the best plant genotypes, microbial strains, fertilizers, and soil conditioners for these objectives. Notably, CRISPR/Cas9 technology could offer potential in increasing plant resistance to highly toxic heavy metals which will further expand the plant species.

From former studies have revealed that the plants under the stress from heavy metals, are compelled to alter their molecular, biochemical and even physiological mechanisms in order to continue grow and develop healthily [88]. With modern biotechnological tools, stress responsive genes of plants like beans which transcribed and translated their proteins and metabolites under heavy metal stress can be now genetically enhanced for putative tolerance. The features of the CRISPR/Cas system present an interesting possibility to increase phytoremediation capabilities of plants. Sequencing data from model phytoremediation plants include Arabidopsis thaliana, red sandalwood, brown algae, mustard, and mustard greens which could be used to isolate and ascertain genes associated with metal stress tolerance. The CRISPR/Cas system can also induce activation for the metal accumulation, degradation, stabilization and filtration inside the rhizosphere, which can be related to the stimulation of the these mechanisms for increasing the overall stress tolerance of the plant [96].

Another type of CRISPR/Cas9 technology includes creating low–cadmium accumulating rice lines through the gene OsNRAMP5 knock down without a significant loss in yield. More recently the use of CRISPR/Cas9 system has been applied to generate OsNRAMP5 knockout rice plants for enhancing cadmium tolerance. But genetic changes in transformed plant colonies may be random and could be off by upto 50 %, in other words, only 20–50 % reflecation of desired mutations would be expected [97]. To the best of the author's knowledge, the specific mechanisms responsible for the life-threatening effects of some Cas9 induced mutations remain unknown. Climate change is another environmental factor that has led to developmental changes affecting plant production thus food security in the world. So, with increased importance of utilizing plants through advanced form of biotechnology, the improvement through genetic modification is of utmost significance. In this way, specific genes and nucleases of the CRISPR/Cas complex allow increasing plants’ tolerance to abiotic stresses. Despite the necessity to evaluate the reliability of some investigations because of the issues in study design, data gathering, and statistical analysis, the potential approaches to improve plant tolerance to heavy metal stress are under development, including synthetic promoters and gene control systems [89]. The activation of heavy metal-induced genes, transcription, factors, and metalloproteinases could be useful for enhancing the plant resistance and phytoremediation. Thus, the subsequent studies should incorporate the diverse strategies and tag the useful genes for abiotic stress tolerance on favorable crop genotypes.

#### 2.2.10 Nano-treatment soil restoration solution

Recently, nanotechnology has been seen as beneficial in many different fields. Nanoparticles (NPs) exhibit several fundamental and encouraging properties because of their versatility in a variety of fields [98]. Reactive NPs are used in remediation technologies to convert and detoxify pollutants [99].There are variations in the ways that different nanomaterials are used for environmental cleanup due to their unique properties. For example, polymer-based materials are the most successful at removing chemical pollutants such as nitrate, manganese, arsenic, and other heavy metals [100]. The surface of the nanoparticles is mainly conjugated with polymers like starch, carboxymethyl cellulose, and various collagens to increase their stability and stop them from accumulating [101]. This is because starch molecules provide a steric barrier that keeps accumulation from happening [102].

For example, a previous work examined the efficacy of starch-conjugated magnetite nanoparticles for immobilizing arsenate (AS) in the *in-situ* remediation of contaminated soil [103].In addition to treating environmental pollutants, they are employed in the detection of many pollutants, including lead, mercury, cadmium, chromium, and selenium [104].

At the nanoscale, Particles of zerovalent iron are inexpensive, non-toxic electron donors that have a faster rate of action when cleaning contaminated soil. Numerous studies were conducted employing nZVI to clean contaminated soil after trichloroethylene was initially eliminated using zero-valent iron nanoparticles [77]. For instance, it was found that the combined effects of polyvinylpyrrolidone (PVP) and nZVI attached to treat soil contaminated with trichloroethylene. The results were convincing, demonstrating that they could eliminate 85 % of TCE by using PVP-nZVI [105].

When microorganisms and biologically generated NPs are employed together, nanoremediation becomes a more ecologically friendly and sustainable process [106]. When iron nanoparticles (NPs) derived from algae were combined with algal cell biomolecules, they demonstrated superior stability, increased reactivity, and effective removal of harmful contaminants from the environment. Conversely, the biogenic function of *Lysinibacillus sphaericus* in the synthesis of magnetic oxide nanoparticles (NPs) is meant to eliminate Cr (VI) pollution from the environment [107]. Studies have shown that certain nanoparticles can generate reactive oxygen species (ROS) in biological systems, leading to oxidative stress in organisms, including plants and microorganisms, which are crucial for maintaining soil health. Furthermore, the stability and degradation behavior of nanomaterials are critical factors that determine their environmental fate. In some cases, engineered nanomaterials can aggregate or undergo surface modifications in natural environments, potentially reducing their reactivity but increasing their persistence [108].

To mitigate these risks, researchers are exploring the use of biodegradable or eco-friendly nanomaterials that degrade into non-toxic byproducts after their remediation function is complete. Additionally, employing containment strategies or recovering nanomaterials after use can minimize their environmental impact. Future research should prioritize the lifecycle assessment of nanomaterials used in remediation, ensuring that their benefits outweigh the risks of secondary pollution.

## 3 Conclusion

There are serious health and environmental hazards associated with heavy metal pollution of soil. Advanced techniques like bioagmutation, CRISPR-mediated gene editing, and nano remediation provide intriguing alternatives, notwithstanding the drawbacks of conventional procedures. A number of variables, such as site features, contamination levels, and intended results, influence the selection of the remediation technique. A thorough evaluation is essential to choosing the best and most sustainable strategy for each unique site.

## Future prospects

Advancements in genetic engineering and the use of microbial communities present promising avenues for enhancing pollutant degradation. Long-term effectiveness, scalability, and cost-efficiency will be key factors driving future developments. Moreover, integrating smart monitoring systems and AI-based approaches for real-time assessment will enable better management and timely interventions in the cleanup of polluted soils. Collaborative interdisciplinary research will be essential to realize these transformative solutions.

## CRediT authorship contribution statement

**Khadija Malik:** Writing – review & editing, Writing – original draft, Visualization, Validation, Supervision, Formal analysis, Data curation, Conceptualization. **Ashja Iftikhar:** Writing – review & editing, Writing – original draft, Visualization, Validation, Resources, Formal analysis, Data curation, Conceptualization. **Quratulain Maqsood:** Writing – review & editing, Writing – original draft, Visualization, Validation, Resources, Investigation, Formal analysis, Data curation, Conceptualization. **Muhammad Rizwan Tariq:** . **Shinawar Waseem Ali:** .

## Declaration of competing interest

All the authors of the article have no conflict of interest to disclose.

## Data Availability

Data will be made available on request.
